# Necrotizing Enterocolitis in a 34-Week Premature Infant with COVID-19

**DOI:** 10.1155/2021/1442447

**Published:** 2021-12-23

**Authors:** Mary K. Mannix, Danielle Blood, Oscar G. Gomez-Duarte, Lauren Davidson

**Affiliations:** ^1^Division of Infectious Diseases, Department of Pediatrics, University at Buffalo, the State University of NY, Buffalo, NY, USA; ^2^Sister's Hospital, Catholic Health System, Buffalo, NY, USA

## Abstract

Coronavirus disease 2019 (COVID-19) is a viral respiratory infection caused by the Severe Acute Respiratory Syndrome Coronavirus 2 (SARS-CoV-2). While SARS-CoV-2 is a leading cause of morbidity and mortality in older adults, COVID-19 also affects newborn infants in nurseries and the Neonatal Intensive Care Units (NICUs). The majority of infected neonates are believed to acquire SARS-CoV-2 by horizontal transmission, and most of them have asymptomatic or mild symptomatic infections. In rare cases, infants with COVID-19 may have severe complications resulting in death. We report a case of COVID-19 in a premature neonate born at 34 weeks gestational age who presented with hypothermia and respiratory distress and subsequently developed clinical and radiological signs of necrotizing enterocolitis (NEC). The neonate received medical management, including antibiotics, suspension of gastric feeds, and intensive NICU support. The neonate's clinical condition improved without surgical intervention, and after 10 days of antibiotics and gradual reestablishment of gastric feeds, patient health condition returned to normal, and weeks later, he was discharged home. COVID-19 in infants is frequently asymptomatic or associated with mild disease, and in rare cases, it may be associated with severe gastrointestinal complications including NEC.

## 1. Introduction

Coronavirus disease 2019 (COVID-19) is a viral respiratory infection caused by the Severe Acute Respiratory Syndrome Coronavirus 2 (SARS-CoV-2), a betacoronavirus, that has spread worldwide reaching pandemic proportions [1]. The COVID-19 pandemic is already a leading cause of morbidity and mortality worldwide with an estimated number of deaths above 3 million according with the John Hopkins dashboard (https://coronavirus.jhu.edu/map.html). The number of deaths due to COVID-19 in the US is the highest in the world and reaching 600,000. Information on the effect of COVID-19 among newborn infants indicates that the majority of infected infants, including neonates in nurseries and Neonatal Intensive Care Units (NICUs), are believed to acquire SARS-CoV-2 by horizontal transmission, and most of them have had asymptomatic or mildly symptomatic infections [2, 3]. Extrapulmonary manifestations of COVID-19 or of multisystem inflammatory disease in children associated to COVID-19 (MIS-C) include gastrointestinal symptoms, and diarrhea, vomiting, and abdominal pain are the most common manifestations [4, 5]. Limited information is available on severe gastrointestinal disorders among premature and full-term newborns. The objectives of this case report are to describe a case of a 34 weeks gestational age premature infant born diagnosed with COVID-19 and necrotizing enterocolitis (NEC), shortly after birth, and to briefly review the literature on the role of COVID-19 on gastrointestinal disorders in pediatrics.

## 2. Case Presentation

A 27-year-old G1P0 mother presented to the hospital in preterm labor with preterm premature rupture of membranes (PPROM) at 33 1/7 weeks gestation. Pregnancy was complicated by the mother with a history of asthma and obesity. Prenatal laboratory results included HIV negative, RPR nonreactive, hepatitis B negative, rubella immune, GC/*Chlamydia* negative, and group B streptococci negative. Upon admission to the labor and delivery unit, the mother tested positive for SARS-CoV-2 by routine nasopharyngeal polymerase chain reaction (PCR) screening. The mother denied exposure to COVID-19 sick contacts, but did work in a community urgent care center (ambulatory walk-in clinic). The mother did not report any respiratory or nonrespiratory symptoms from her COVID-19 infection during or following her hospital stay.

Following admission for PPROM, the mother was placed on cardiotocographic fetal monitoring in the labor and delivery unit. The fetal heart tracing was reassuring with a reactive fetal heart tracing. Fetal ultrasound showed the fetus in cephalic presentation with an estimated fetal weight, at the 30th percentile for age, of 2,031 g. The mother was given a course of betamethasone to facilitate fetal lung maturity, and she was started on prophylactic azithromycin and ampicillin, due to risk of infection secondary to PPROM. The mother was kept on the antepartum service until she reached 34 weeks gestation with reassuring daily biophysical profiles and unremarkable serial complete blood count (CBC) monitoring.

**Figure 1:**
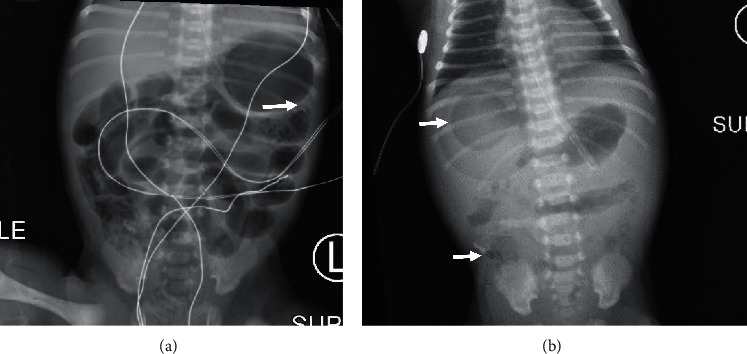
Abdominal x-rays of the premature infant. (a) Image obtained at 30 h of age. Arrow indicates the area of pneumatosis intestinalis. (b) Image obtained at 36 h of age. Upper arrow points to one area of pneumoperitoneum, and the lower arrow points to an area of pneumatosis intestinalis.

A planned induction of labor was started at 34 weeks gestation which resulted in a spontaneous vaginal delivery of a healthy male newborn with APGAR scores of 8 at 1 minute and 8 at 5 minutes and weighing 2,065 g. The placenta, evaluated by pathology, showed a small intraparenchymal hematoma and a small focal infarction. The mother did not have contact with her newborn postnatally and was not permitted to visit the NICU until 10 days from her positive COVID-19 testing, per hospital policy and Centers for Disease Control and Prevention (CDC) guidelines. The neonate was admitted to the NICU immediately following delivery. He was breathing room air without tachypnea or increased work of breathing and was started on oral feeds of expressed breast milk and supplemented with formula when breast milk was not available. CBC with differential on admission was stable without a left shift (Table 1), and a blood culture sent was negative at 48 hours. Upon admission to the NICU, the neonate was empirically started on intravenous ampicillin and gentamicin infusions, per suspected neonatal sepsis protocol, due to PPROM for 6 days prior to delivery. At approximately 15 hours of age, the newborn patient had tachypnea with a respiratory rate of 80 breaths per minute. At approximately 20 hours of life, the neonate had temperature instability with axillary temperatures as low as 35.8 C°. The first SARS-CoV-2 PCR test, drawn per CDC recommendations at 24 hours of age, was negative. At approximately 25 hours of age, the neonate had evidence of feed intolerance with gastric aspirates of up to 6 mL of undigested milk and an abdominal X-ray (AXR) showing gaseous distension. On physical exam, the abdomen remained soft with bowel sounds present in all 4 quadrants and no other focal findings. Feeds were continued under close observation. At approximately 30 hours of life, the newborn had a feed residual of 19 mL (90% of feed volume), and a follow-up AXR showed signs of pneumatosis intestinalis (Figure 1(a)). On suspension on NEC, the patient's gastric feeds were immediately discontinued, parenteral nutrition was initiated, and ampicillin and gentamicin were continued. Repeated CBC with differential showed an immature to total neutrophil (iT) ratio of 0.39 (normal < 0.16) (Table 1) and a C-reactive protein (CRP) of 77 mg/L (normal range 1.0–5.0 mg/L). At 36 hours of age, the AXR confirmed pneumatosis intestinalis, and in addition, it revealed pneumoperitoneum in the right upper quadrant (Figure 1(b)). Based on the abovementioned clinical, laboratory, and radiological findings, a diagnosis of NEC was established. Surgical service was consulted, and medical management was continued with modifications. First, metronidazole was added to the antibiotic regimen in place to improve anaerobic bacterial coverage. Also, a replogle tube was placed to low intermittent wall suction to relieve gastrointestinal pressure. The second SARS-CoV-2 PCR test drawn at 48 hours of age was positive. On day 5 of antibiotics therapy, pneumatosis intestinalis and pneumoperitoneum resolved on AXRs and CRP trended down to 23.3 mg/L. Surgical intervention was not required. The patient remained on triple antibiotic therapy for 10 days. By the end of the antimicrobial treatment, respiratory and gastrointestinal function returned to normal and gastric feeds were gradually resumed to baseline levels. The neonate recovered from NEC with medical management, and he was later discharged to home.

## 3. Discussion

Case series of COVID-19 in neonates have identified a variety of clinical presentations from asymptomatic to severe disease, most often presenting with respiratory distress [6]. The National Perinatal COVID-19 Registry has no reports of COVID-19-related neonatal deaths, and only 2% of neonates have tested positive after being born to a mother infected with COVID-19 [7]. Our case describes a 34-week premature infant born to a mother positive for SARS-CoV-2 by PCR who developed signs and symptoms of NEC at 30 hours of age and was subsequently diagnosed with COVID-19 at 48 hours. The neonate was tested for SARS-CoV-2 due to maternal infection, as recommended by CDC guidelines for neonates born to COVID-19-positive mothers.

Necrotizing enterocolitis is a significant cause of morbidity and mortality among infants in the NICU. Although necrotizing enterocolitis continues to afflict neonates of all sizes and gestational age, it is much more commonly seen in infants born less than 1500 g in weight and less than 28 weeks in gestation [8]. The pathophysiology of NEC is multifactorial, resulting in acute inflammation and invasion of the intestinal wall. Classic risk factors for NEC include premature rupture of membranes, extreme prematurity, very-low-birth-weight (VLBW) infants, exposure to medications including antibiotics, and exposure to formula [9]. A study conducted by Sharma et al., including over 200 infants with NEC, found that the mean age of onset was around 20 days of life [10]. Our patient was born at 34 weeks gestation, was over 2,000 g at the time of birth, and had signs of NEC at 30 hours of life which is much earlier than frequently seen with NEC. There is a likelihood that COVID-19 in this premature infant contributed directly or indirectly to NEC. Only one case of an NEC-like disorder was reported in the literature in a 7-week-old term male with confirmed pneumatosis intestinalis who was also positive for COVID-19 [11].

Coronavirus infection in human subjects is not limited to the respiratory tract. There was a reported cluster of NEC cases associated with infections due to coronavirus-like agents in the early 1980s. Histopathology evaluation of intestinal specimens revealed coronavirus-like particles within intracytoplasmic vesicles of damaged mucosal cells of the small intestine, appendix, and colon [12, 13]. In addition to NEC, there have been reports of gastrointestinal disorders associated with COVID-19 or MIS-C in pediatric patients that are manifested with nausea, vomiting, diarrhea, and abdominal pain [4, 5, 14]. Appendicitis in children has been associated with acute presentations of COVID-19 [15]. Furthermore, the severe acute respiratory syndrome caused by the SARS coronavirus in China back in 2003 was associated with gastrointestinal disorders including diarrhea [16]. How SARS-CoV-2 may induce severe inflammation leading to NEC in a small number of infants is a question that remains unanswered. The gut microbiome may be the link between COVID1-19 and gastrointestinal disorders [17], yet it is unclear whether gut microbiome dysbiosis may explain the severe gastrointestinal manifestations during COVID-19 in some patients [18]. Overall, it is likely that acute inflammation in the gastrointestinal track during or after SARS-CoV-2 infection in association with gut microbiome changes may lead to severe gastrointestinal disorders in infants, including NEC. One limitation of this report was the lack of SARS-CoV-2 PCR testing of the infant's stool. A positive result would have corroborated the association of gastrointestinal SARS-CoV-2 infection with NEC in this infant.

## 4. Conclusions

This was a formula-fed premature newborn infant who shortly after birth had gastric feed intolerance and AXR imaging consistent with NEC. This case of NEC is unusual because the age of onset was too early, the patient was not a very-low-weight premature infant, and the infant had no low gestational weight. The fact that the neonate had COVID-19 at the time NEC was diagnosed suggests that SARS-CoV-2 infection was likely associated with this severe gastrointestinal disorder.

## Figures and Tables

**Table 1:** Infant's complete blood cell count (CBC) values at two time points after birth.

Parameters	Values DOL1 0	Values DOL1 2	Reference values
WBC	12.3	8.3	7.0–30.0 × 10e3/*μ*L
RBC	5.47	4.71	4.0–7.0 × 10e6/*μ*L
HGB	20.3	17.4	14.5–22.5 g/dL
HCT	58.2	50.5	45.0–67.0%
MCV	106.5	107.2	95.0–121.0 fL
MCH	37.1	37.0	31.0–37.0 pg
MCHC	34.9	34.6	29.0–37.0 g/dL
RDW	15.9	16	13.0–18.0%
PLTS	353	307	150.0–300.0 10e3/*μ*L
MPV	8.4	10.0	29.0–37.0 g/dL
Neutrophils (%)	73	27	30–60%
Bands (%)	0	17	0–9%
Lymphocytes (%)	18	43	25–50%

DOL: day of life.

## Data Availability

The data that support the findings of this study are available from the corresponding author upon reasonable request.
